# Evaluating suitability of source water for a proposed SWRO plant location

**DOI:** 10.1016/j.heliyon.2019.e01119

**Published:** 2019-01-09

**Authors:** Mohamed O. Saeed, Mohammed A. Al-Nomazi, Ahmed S. Al-Amoudi

**Affiliations:** Desalination Technologies Research Institute, Saline Water Conversion Corporation, PO Box 8328, Al-Jubail 31951, Saudi Arabia

**Keywords:** Ecology, Microbiology

## Abstract

SWRO membranes are characterized by their ability to reject particles of larger than 0.001 μm diameter and approximately 200 molecular weight. This indicates the selectiveness of the SWRO membranes and the relative ease by which they can be clogged or fouled. For this reason, membrane filtration needs the cleanest possible feed water. Without extensive feed water purification, the reverse osmosis (RO) technology of simple filtration under pressure could be fraught with problems. The quality of source water determines the pretreatment regimen and consequently success or failure of a SWRO plant. The present study evaluated the suitability of source water for a proposed SWRO plant on the Gulf coast of Saudi Arabia.

Various physico-chemical and biological parameters were assessed: temperature, pH, salinity, conductivity, total dissolved solids (TDS), silt density index (SDI), turbidity, total suspended solids (TSS), total hardness, total alkalinity, total organic carbon (TOC), dissolved carbohydrates and proteins, dissolved oxygen (DO), biochemical oxygen demand (BOD), chlorophyll-a, bacterial count, major ions, and trace metals. With the exception of total suspended solids, chemical and physicochemical variables measured in this study had concentrations typical of the Arabian Gulf water and seawater in general. Average TSS values were slightly higher than those reported for Gulf coastal waters, and more importantly they were highly variable. This variability may result in episodes of filtration problems for the SWRO plant. The plant should seek deeper sea water a distance from shore as the location of its intake. The study allowed recommendations for treatment options to assure more successful operation of the plant.

## 1 Introduction

Over the years and until quite recently, seawater desalination witnessed thermal processes take precedence over seawater desalination. However, in the last few years, seawater reverse osmosis (SWRO) desalination technology has undergone a remarkable transformation and gained widespread acceptance, which is evident from SWRO's increasing share of the total installed/contracted seawater desalination capacity. This publication describes an assessment of seawater quality at a site on the Gulf coast of Saudi Arabia so as to evaluate its suitability for a SWRO plant. The site is located on the coast of Al-Khafji City in the eastern region of Saudi Arabia (location: latitude of 28–26 N and a longitude of 48–30 E). The near shore is mostly rock formation covered by a thin layer of sand with scattered sea grass and macroalgae [1]. The off-shore zone of the study area is close to the Saudi-Kuwaiti border and close to the location and activities of the Saudi-Kuwaiti Al-Khafji Joint Operations (KJO) Concession. The location is close to the site of oil and gas production platforms, submerged pipelines, marine oily water discharges, decommissioned pipelines, as well as related equipment and facilities [1]. In addition, the site is open for discharges from a multi stage flash (MSF) desalination plant.

The Gulf is a unique and heavily used body of water. Its physical configuration poses distinctive challenges [2]. It is a shallow, semi-enclosed body of water with little input from precipitation or inflow from rivers. This configuration gives Gulf water its unique characteristics. Gulf seawater has a TDS concentration of approximately 43,800 mg/l or higher, typically higher than TDS in normal seawater where the concentration is about 35,000 mg/l. The average ionic ratio of Gulf seawater to that of normal seawater is approximately 1.25:1, and consequently the ionic concentration is proportionally higher in Gulf seawater than in normal seawater. Summer water temperature can exceed 36 °C while winter water temperature can fall below 15 °C [2].

The Gulf is subject to the combined effects of economic and population growth with high levels of industrial activity, e.g., desalination, infrastructure, coastal construction and coastline alterations [3]. In addition, the Gulf is subject to pollution from oil production and maritime transport associated with the oil industry. Maritime transport can create unexpected changes in the biotic community by introducing new species to the Gulfs' unique habitat [4].

The main objective of this study is to evaluate the quality of open seawater at the proposed site for a period of 12 months so that optimum pretreatment for a proposed seawater reverse osmosis plant could be identified.

## 2 Methodology

### 2.1 Depth profile study

At the beginning, a depth profile assessment was carried out by analyzing surface and bottom water samples from an area of approximately 10 m depth and 2000 m off shore. When no differences in water quality were detected (see Results anddiscussion), water samples were taken from 1–2 m below the surface in a water column of approximately 5 m depth at a distance of approximately 700 m off shore for the whole study.

### 2.2 Physico-chemical and biological parameters

Water quality evaluation involved collecting and analyzing of seawater samples for 12 months, including all seasons, between 2014 and 2015. The parameters for water quality assessment included the following physical, chemical and biological parameters: temperature, pH, salinity, conductivity, total dissolved solids (TDS), silt density index (SDI), turbidity, total suspended solids (TSS), total hardness, total alkalinity, total organic carbon (TOC), dissolved carbohydrates, dissolved proteins, dissolved oxygen (DO), biochemical oxygen demand (BOD), chlorophyll-a, bacterial count, as well as major ions, and trace metals.

Methodology used for seawater quality analysis in the present study with relevant notes on the analytical methods used is summarized in Table 1. The procedures were detailed in standard methods [5] ad a manual of seawater analysis [6]. The research was carried out from July 2014 to June 2015. The study comprised monitoring and laboratory work. Field monitoring included the direct measurement of pH, temperature and salinity, the collection of water samples to determine other parameters, i.e., conductivity, turbidity, total suspended solids (TSS), silt density index (SDI), total dissolved solids (TDS), hardness, alkalinity, ions, trace metals, total organic carbon (TOC), chlorophyll, biochemical oxygen demand (BOD_5_), and bacteriological analyses. Sampling was carried out on a fortnightly and monthly basis. Dissolved carbohydrates and proteins were determined once to assess the effect of chlorination on their availability. A motorized boat was used in field trips, and the collected water samples were kept on ice and analyzed immediately upon arrival at the laboratory. There were a total of 20 cruises covering all four seasons (5 each during summer, autumn, winter, and spring) during the study period.

**Table 1:** Techniques and methods employed in seawater analyses.

Parameters	Techniques	Methods
pH	Calibrated pH meter	-
Temperature (°C)	Mercury thermometer	-
Salinity (‰)	Temperature-compensated refractometer	-
Conductivity (μS/cm)	Conductivity by probe	Standard methods (SM) 2510 B [5]^a^
Turbidity (NTU)	Nephelometric method	SM 2130 B
Total suspended solids (TSS)	Filtration and drying	SM 2540 D
Silt density index (SDI_15_ units)	0.45-μ Pore size filter with SDI Manifold	ASTM^b^ D4189-95
Total dissolved solids (TDS, mg/l)	Drying @ 180 °C to a constant weight.	SM 2540 C
Hardness (mg/l)	Titrimetric, EDTA	SM 2340 C
Alkalinity (mg/l)	Titration	SM 2320 B
Chloride (Cl^−^), mg/l	Potentiometric titration with silver nitrate	SM 4500Cl D
Sodium (mg/l)	Inductively coupled plasma-atomic absorption emission spectroscopy (ICP-OES)	SM 3120 A
Calcium (Ca), mg/l	ICP-OES	SM 3120 A
Magnesium (Mg), mg/l	ICP-OES	SM 3120 A
Potassium (K), mg/l	ICP-OES	SM 3120 A
Barium (Ba), μg/l	ICP-OES	SM 3120 A
Strontium (Sr), μg/l	ICP-OES	SM 3120 A
Boron (B), μg/l	ICP-OES	SM 3120 A
Iron (Fe), μg/l	ICP-OES	SM 3120 A
Copper (Cu), μg/l	ICP-OES	SM 3120 A
Cobalt (Co), μg/l	ICP-OES	SM 3120 A
Manganese (Mn), μg/l	ICP-OES	SM 3120 A
Zinc (Zn), μg/l	ICP-OES	SM 3120 A
Nickel (Ni), μg/l	ICP-OES	SM 3120 A
Vanadium (V), μg/l	ICP-OES	SM 3120 A
Selenium (Se), μg/l	Atomic absorption–vapor generation analyzer technique	SM 3114 B
Arsenic (As), μg/l	Ditto	SM 3114 B
Lead (Pb), μg/l	Atomic absorption-graphite tube atomizer	SM 3111 B
Chromium (Cr), μg/l	Ditto	SM 3111 B
Cadmium (Cd), μg/l	Ditto	SM 3111 B
Mercury (Hg), μg/l	Atomic absorption – cold vapor generation analyzer technique	SM 3112 B
Fluoride (F^−^), μg/l	Ion chromatography	SM 4110 A
Sulfate (SO_4_^2−^), mg/l	HACH	SM 4110 A
Nitrate (NO_3_^−^), μ/l	−	SM 4110 A
Phosphate (PO_4_^3−^), μ/l	−	SM 4110 A
TOC (Total Organic Carbon, mg/l)	Total organic carbon in water	SM 5310A
Dissolved sugars (mg/l)	Acid hydrolysis	Parsons, *et al.*[6]
Dissolved proteins (mg/l)	Folin-ciocalteu reagent	Parsons, *et al.*[6]
Biochemical oxygen demand (BOD_5_, mg/l)	Initial and final dissolved oxygen	Winkler
Chlorophyll-a (μ/l)	Acetone extraction	Parsons, *et al.*[6]
Total viable bacterial count (Colony-Forming Units/ml)	Heterotrophic plate count (pour plate method in marine agar)	SM 9215 B
Fecal coliform	Fecal coliform procedure	SM 9221 E

a Numbers in squared parenthesis refer to references.

b American Society for Testing sand Materials.

Initial (0-h) and final (48-h) counts were used to calculate bacterial generation time (doubling time) in hours [7]. The bacterial generation time indicates the speed of bacterial multiplication, which reflects the status of nutrient availability in the water. Source water with a high bacterial multiplication capacity poses a greater threat to membrane fouling than nutrient- deficient waters.

### 2.3 Chlorination disinfection

The plant will use chlorination for disinfection to protect the intake structures from marine shell fouling. An experiment was carried out to assess the effect of source water chlorination on SDI, TSS and concentrations of dissolved organic nutrients (carbohydrates and proteins). Analyses of these parameters were carried out on triplicate water samples following procedures outlined in Table 1.

Samples of coastal water were filtered through a 0.3-mm nylon mesh to remove debris, and passed through 5.0-μm membrane filters to remove micro-organisms. One portion of each water sample was analyzed for the above-mentioned parameters after filtration and the other portion was analyzed after filtration and chlorination. Chlorine (household bleach) was added until a residual of 0.3 mg/l was reached. After 20 min, chlorine was removed by the addition of sodium metabisulfite (SBS).

## 3 Results and discussion

### 3.1 Depth profile study

Results of the depth profile study showed no significant difference between measured water quality parameters in bottom and surface water samples at a depth of 10 m and a distance of 2000 m off-shore (Table 2). However, total suspended solids concentration in the surface was more variable than in bottom. Similar results were reported from Jubail and Jeddah coastal waters in the vicinity of the Saline Water Conversion Corporation's desalination and power plants [2, 8]. Thereafter, sampling was restricted to a location of approximately 5-m depth and 700 m offshore. Shallow coastal waters are well mixed and surface samples seem to represent the water column. The water quality variation in temperature, salinity, turbidity, and dissolved oxygen between surface and a depth of 90 m in the Red Sea would not have a significant impact on a SWRO plant operation. There is an overall decrease in algae, bacteria, TOC, TEP, and biopolymer concentration with depth, which could reduce the potential for membrane biofouling, but not to a large degree [9]. Because of variability of TSS in surface water and because shallower water near shore is more easily disturbed than deeper off shore water, the plant is advised to source water from well below the surface at the off shore location.

**Table 2:** Concentration of certain water quality parameters in bottom and surface Gulf coastal water at Khafji, Saudi Arabia (n = 3).

Parameters^a^	10-m deep water^b^		5-m deep water^c^
	1 m below surface	1 m above bottom	1 m below surface
TSS (mg/l)	15.0–21.1	16.2–19.0	16.0–21.4
SDI_15_	5.7–5.9	5.9–6.1	5.8–6.3
TOC (mg/l)	2.2–2.4	2.1–2.3	1.9–2.3
BOD_5_ (mg/l)	0.6–0.8	0.5–0.7	0.6–0.9
Chlorophyll (μg/l)	1.2–2.4	0.9–1.8	1.1–2.0
Salinity (‰)	41.2–42.0	40.9–41.7	40.0–42.5
pH	8.1–8.2	8.1–8.2	8.1–8.2

a For the same parameter and at the tree locations means are not different (Paired t-tests (P < 0.05).

b 2000 m off-shore.

c 700 m off-shore.

Results of the near shore analyses are presented in Tables 3, 4, 5, and 6. Parameters which are more likely to have a direct impact on the operation of a SWRO plant are segregated into seasons (Table 3) in order to predict possible seasonal filtration/operational difficulties that may face a SWRO plant. Other stable chemical parameters e.g. major ions and the widely variable trace metals (Tables 4 and 5) are not segregated into seasons. Effect of chlorination on TSS, SDI, dissolved carbohydrates and dissolved proteins is presented in Table 6.

**Table 3:** Summary of water quality parameters in the Gulf coast water at Khafji, Saudi Arabia during the four seasons.

Parameters	Summer			Autumn			Winter			Spring		
	Min	Max	Mean^a^	Min	Max	Mean^a^	Min	Max	Mean^a^	Min	Max	Mean^a^
Temperature (°C)	31.5	34.0	32.5	21.4	27.5	24.9	13.0	18.5	16.3	21.5	30.4	25.9
Salinity (‰)	41.8	42.0	41.0	40.9	41.4	41.1	40.4	41.6	41.0	41.0	42.7	41.5
Conductivity (μS/cm)	61600	63400	62850	61400	61900	61600	61200	62500	61642	61500	63400	62450
TDS (mg/l)	42800	47150	45122	44000	46460	45690	43500	46900	45082	45295	45285	45290
Turbidity (NTU)	0.20	0.88	0.53	0.85	9.20	3.78	0.45	6.97	2.82	0.55	1.00	0.78
TSS(mg/l)	7.0	25.3	15.5	7.0	46.7	22.9	15.5	22.5	20.2	20.0	22.0	21.0
SDI_15_	5.2	6.5	5.9	6.1	6.4	6.3	5.1	6.3	5.7	5.6	6.2	5.9
pH	8.2	8.2	8.2	8.1	8.2	8.2	8.1	8.2	8.1	8.2	8.2	8.2
Total alkalinity (mg/l)^b^	126.0	128.0	126.5	125.0	126.0	125.5	125.0	127.0	126.5	126.0	132.0	127.8
Total hardness (mg/l)^b^	7120	8052	7761	7840	8159	8033	6648	8648	8124	8140	8205	8173
TOC(mg/l)	1.8	2.7	2.4	2.0	2.9	2.4	2.1	2.6	2.3	2.1	2.1	2.1
BOD_5_ (mg/l)	0.2	1.2	0.7	0.3	1.4	0.9	0.8	1.2	1.0	0.6	0.6	0.6
Chlorophyll (μg/l)	0.5	1.9	0.6	1.2	4.2	2.8	4.3	4.4	4.3	3.4	4.8	4.3

a Relative standard deviation less than 5%.

b CaCO_3_equivalant.

**Table 4:** Concentration of major ions in Gulf coast water of the study area at Khafji, Saudi Arabia.

Parameter	Concentration (Proposed location)	Concentration (Normal seawater)
Chloride (Cl^−^), mg/l	23500–25000	18980 [21]^a^/19110 [22]
Sodium (Na^+^), mg/l	12150–14690	10,556 [21]/10,787 [22]
Calcium (Ca^2+^), mg/l	434–536	400 [21]/412 [22]
Magnesium (Mg^2+^), mg/l	1436–1776	1262 [20]/1,267 [22]
Potassium (K^+^), mg/l	388–543	380 [21]/398 [22]
Sulfate (SO_4_^2+^), mg/l	3216–3467	2649 [21]/2700 [24]
Nitrate (NO_3_^−^), μg/l	5.1–7.0	0.06–30 [23]
Phosphate (PO_4_^3+^), μg/l	1.05–5.50	0.00–300 [23]
Fluoride (F^−^), mg/l	0.10–1.00	1.0–1.3 [23, 24]

a Numbers in squared parenthesis refer to references.

**Table 5:** Trace metal concentrations in Gulf coastal water at Khafji, Saudi Arabia.

Metals	Concentration range (Khafji)	Concentration in gulf water	Concentration in sea water
Barium (Ba), μ/l	10.00–12.00	<50 [25]	21–30 [24, 21]^a^
Strontium (Sr), mg/l	5.00–10.00	9.5–15 [25, 26]	8.1 [24], 13 [21], 90 [23]
Boron (B), mg/l	4.6–5.2	4.9–6.1 [27]	2.1–4.5 [23]
Iron (Fe), μg/l	0.85–1.63	0.15–8.63 [1]	0.056–3.4 [23, 24]
Copper (Cu), μg/l	0.20–7.40	0.19–10.49 [1]	0.03–0.90 [23, 24]
Cobalt (Co), μg/l	Not detected	0.00–0.63 [28]	0.0006–0.39 [23, 24]
Manganese (Mn), μg/l	0.00–0.10	0.00–1.24 [1, 29]	0.011–0.40 [23, 24]
Zinc (Zn), μg/l	1.00–5.00	0.81–118 [1, 29]	0.003–5.0 [23, 24]
Nickel (Ni), μg/l	0.95–1.55	0.18–1.78 [1]	0.12–6.6 [23, 24]
Vanadium (V), μg/l	0.06–1.9	0.03–2.32 [1, 29]	1.90–1.02 [23, 24]
Selenium (Se), μg/l	0.7–1.4	–	0.04–0.90 [23, 24]
Arsenic (As), μg/l	1.00–4.00	0.85–4.46 [1, 28]	1.12–2.6 [22, 23]
Lead (Pb), μg/l	0.00–0.037	0.008–2.10 [1]	0.01–0.36 [22, 23]
Cadmium (Cd), μg/l	<0.01–0.03	0.02–5.2 [1, 28]	0.0001–0.12 [[22], [23]]
Chromium (Cr), μg/l	0.10–0.13	0.00–4.13 [1, 27]	0.10–0.26 [22]
Mercury (Hg), μg/l	<0.01–0.07	0.001–0.38 [28]	0.0004–0.15 [22, 23]

a Numbers in squared parenthesis refer to references.

**Table 6:** Effect of chlorination on TSS, SDI and Dissolved Carbohydrates and Proteins (n = 3).

Filtered seawater				Filtered and chlorinated seawater			
TSS mg/l	SDI	Carbo hydrates mg/l	Proteins mg/l	TSS mg/l	SDI	Carbo hydrates mg/l	Proteins mg/l
0.6	5.8	0.80	0.20	0.8	6.4	1.6	0.50
0.5	5.5	0.81	0.19	0.6	6.5	1.6	0.50
0.6	5.6	0.82	0.23	0.7	6.4	1.8	0.53
X^−^ ± SD 0.57 ± 0.05^a^	5.63 ± 0.15^b^	0.81 ± 0.01^d^	0.21 ± 0.02^f^	0.70 ± 0.01^a^	6.43 ± 0.06^c^	1.67 ± 0.12^e^	0.51 ± 0.02^g^

^a,b,c,d,e,f,g^For the same parameter means of filtered and filtered and chlorinated seawater with same letter superscript are not different, those with different letter superscripts are different. Paired t-tests (P < 0.05).

### 3.2 Turbidity and total suspended solids (TSS)

Turbidity is a key parameter in pretreatment strategies for operating RO plants. Turbidity values were normally stable, but with occasional abrupt rises associated with rough sea conditions. The lowest turbidity of 0.2 was recorded during summer while the highest turbidity of ≈7 was recorded during winter.

Total suspended solids are important water quality parameters that affect the community structure of an aquatic ecosystem. Suspended solids in seawater may comprise inorganic particles as well as dead or living organic material. They are small particles that float in suspension through the water column. Suspended living organic material, e.g. bacteria and microalgae, are an important food source for the next trophic level, and they may therefore enrich productivity. On the other hand, they may cause light attenuation and limit phytoplankton production. These effects may upset both the food chain and the type and magnitude of a biofouling community, either through macrofouling of plant structures or microfouling of filters and membranes. The TSS values in the feed water intake of Al-Khafji plant were generally high and variable. The minimum TSS value was 7 and the maximum value was 46 mg/l, with the majority of readings in the upper teens and an overall average of 20 mg/l. In general, TSS was more variable in autumn (7.0–46.7 with a mean of 22.9 mg/l) than in winter (15.5–22.5 with a mean of 20.2 mg/l, Table 3). The *Shamal* wind and rain storms are key factors contributing to elevated turbidity and TSS values in winter. Abnormally high TSS values in autumn could be attributed to a localized event involving currents or waves.

TSS values reflect the agitation of seawater by wind, waves and currents. In shallow waters the bottom is easily disturbed, bringing more suspended solids load to the water column with a consequent rise in TSS concentration. The sampling location (in the vicinity of the SWCC Al-Khafji desalination plant) is close to the discharge zone where discharged water may further agitate the water column. The through mixing of the water column is reflected in high dissolved oxygen values (5.5–7.0 mg/l).

The present TSS values were close to those reported at the intake bay of the SWCC desalination and power plants at Al-Jubail, where the plant faces frequent filtration problems [2]. In the Jubail plant, the dual media filter was designed to handle feed water of up to 20 mg/l TSS. This value was set, based on TSS values which were severely underestimated at lower than 1 mg/l. This was thought to allow for a safety margin of about 19 mg/l TSS. However, actual TSS values were found to be much higher and close to the design limit of 20 mg/l, thus allowing only a narrow safety range. As it was, the plant experienced filtration problems.

The expectation is that the new SWRO plant will face filtration problems unless the type of pretreatment and its efficiency are appropriate, e.g. media filters with low pressure membranes and media filtration with DAF system. The DMF should be designed to handle unexpected episodes of high TSS loads. The coast is very shallow even at 700 m off-shore where sampling was carried out. If the plant is to seek deeper water, the intake pit has to be located some 2000 m off-shore, and even at this distance the depth is only about 10 m. An intake tower at this distance would allow withdrawing water from about 3 m above the bottom. To avoid disturbing the water column by an intake pump, the water could flow by gravity through the intake pipe to a coastal receiving tank that act as a sedimentation pond. This arrangement should result in appreciable decrease of the silt load in the preceding filtration system.

### 3.3 Silt density index (SDI)

Good quality source water is essential for the smooth operation of desalination plants, particularly seawater reverse osmosis (SWRO) plants. Even good source water has to be treated to attain a certain level of purity before being fed to desalination membranes.

SDI is the key practical parameter used when assessing the level of feed as well as source water purity. The mean SDI values (15-min SDI) recorded in this study are typical of Gulf water. Mean SDI values ranged from 5.7 in winter to 6.3 in autumn. For example, the average SDI value for untreated Gulf water is 5.9 at Jubail in Saudi Arabia and ≥6.5 at Doha, Kuwait [10]. By comparison, the average SDI values for untreated source water to SWCC SWRO plants located on the Red Sea coast are 4.6 at Haql, Duba, and Umm Lujj in the north, 4.8 at Jeddah in the center and 6.5 at Al-Birk, in the south [10].

**Fig. 1:**
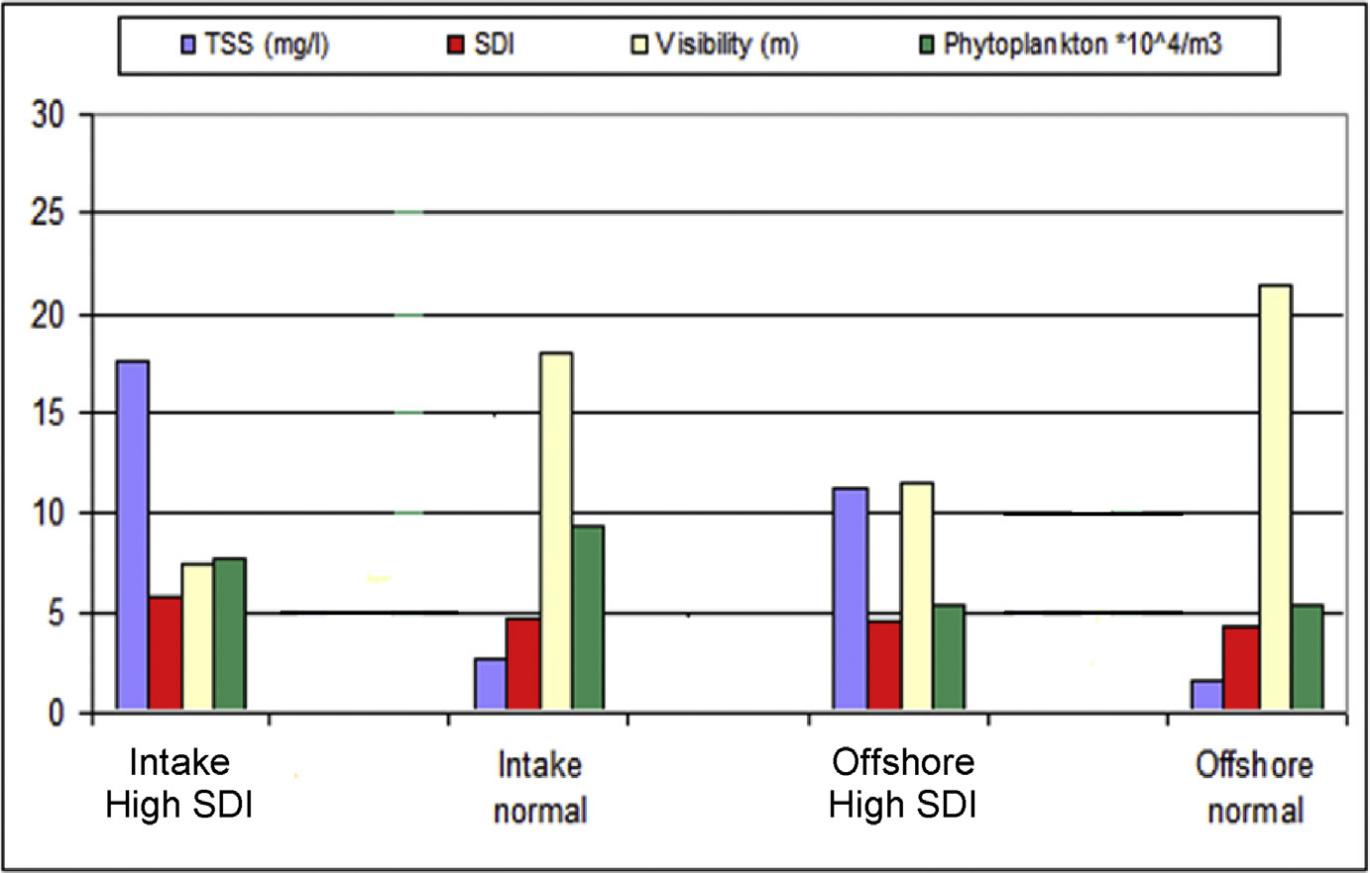
Comparison of total suspended solids (TSS), silt density index (SDI), Secchi disc visibility and phytoplankton density during high and normal SDI periods in coastal (RO intake) and offshore Red Sea water (about 5 km off shore) at the SWCC Jeddah desalination and power plants [8]. Note: The poor relationship between TSS load and SDI values indicating other components like transparent exopolymer molecules affecting SDI values.

In general, the average SDI value is higher in autumn than in the other seasons. The lowest autumn SDI value is higher than the highest SDI value recorded in the other seasons (Table 3). The explanation for this is likely to be climate change and local currents. The highest SDI values recorded in autumn coincided with the highest TSS values. The SWCC Jeddah SWRO plants used to encounter high SDI episodes in autumn (October), and the cause was traced to a local seasonal current that disturbs the bottom sediments [8]. However, the relationship between TSS and SDI is not linear [8] since other components, e.g. transparent exopolymeric (TEP) organic molecules can also contribute to a rise in SDI (Fig. 1). The TEP substances have gained attention as causal agents of membrane fouling and filter plugging. Source water intended for new SWRO plants should be investigated for TEP content. The European Desalination Society (EDS) Conference in Barcelona in 2012, vigorously addressed TEP problems with multiple sessions focusing on the TEP phenomenon. The aforementioned EDS sessions clearly indicated that traditional filtration pretreatment is not efficient in removing TEP molecules, and that ultrafiltration is much more efficient in filtering out TEP molecules. The new plant is thus encouraged to introduce ultrafiltration membranes in pretreatment set-up. Clearly, there are multiple factors governing SDI, and although RO technology may appear to be a simple technique of filtration under pressure, in practice it is fraught with problems. Also, SWRO plant problems tend to be unique: Information gathered when solving a problem at one location seldom becomes useful at another. This is because SWRO operational problems are site-specific. This has led to diversity in pretreatment process design, operation techniques and troubleshooting responses. Localized procedures, therefore, have to be developed and practiced until they become established for a given location.

Carefully tested pilot pretreatment is particularly indicated in seawater RO plants with open coastal water as source water. The need for proven pretreatment becomes essential in plants along the Arabian Gulf shore. The hot climate makes the water temperature conducive to biological growth and membrane fouling. The sea is very shallow, with abundant sunlight, which promotes biological productivity. Shallowness also allows the water column to become easily disturbed. Total suspended solids vary greatly in their concentration due to this combination of high biological productivity and the easy disturbance of the water column. At times, high TSS values create filtration problems and plants may need to be totally shut down until SDI values return to normal. This problem occurs in many plants along the Red Sea and Gulf coasts.

### 3.4 Temperature

Temperature in the study area (Table 3) is typical of the Gulf's temperature profile and within the range reported for the area [1] and for the Arabian Gulf [11]. Along the Arabian Gulf coast, the summer temperature ranges from 30.6 to 36.8 °C, the winter temperature from 11 to 22 °C, the spring temperature from 18.7 to 32.8 °C and the fall temperature from 18.8 to 32.8 °C, while the air temperature never drops below freezing [11]. In general, the seawater temperature in the Arabian Gulf is governed by two main factors; the *shamal* wind in winter and solar radiation in summer [12].

The basic design criteria for a SWRO plant are likely to call for a temperature range of 20 °C –35 °C. At extreme winter temperatures, the RO membranes become tight with a decrease in permeate flux; for this reason, the temperature is normalized. One way to counter the effect of cooler temperatures on membrane performance is to use water from a nearby MSF plant heat rejection section. This hybrid operation will increase the SWRO plan's productivity, but energy costs (e.g. that of pumping) have to be viable.

### 3.5 pH and total alkalinity

pH values remained consistently stable between 8.1 – 8.2 during the study. This falls within the tolerance range for SWRO membranes. pH is a very stable parameter in seawater due to the enormous buffering capacity of seawater. Even during algal blooms with their associated high consumption of carbon dioxide, the pH fluctuates only slightly. Therefore, the pH will always remain basic, and acidification to lower the pH and combat scaling should be practiced as usual.

Alkalinity (also known as “buffer capacity”) is a measure of the capacity of water to neutralize acids. The range of alkalinity in Khafji water samples during the present study was 125.5–132.0 mg/l CaCO_3_ equivalents. This value is slightly higher than that reported for normal seawater, which is in the range of 116.0–125.0 mg/l [13, 14]. This is understandable because alkalinity species (e.g. bicarbonates, carbonates and hydroxides) are comparatively more concentrated in the Arabian Gulf. Therefore, the buffering capacity of the Khafji or Gulf water in general is higher than in normal seas. The capacity to resist pH changes is critical for aquatic life. The ability to neutralize acids makes Gulf water more resistant to acidic discharges, acid rain, etc.

### 3.6 Total dissolved solids (TDS) and salinity

There was not a significant change in salinity values during the study. Salinity varied from 40.4 – 42.7 ‰. Generally, all the observed salinities at the study site fell within those values reported previously in the Arabian Gulf area [12].

TDS is the term used to describe the inorganic salts and small amounts of organic matter present in solution. The principal constituents are usually calcium, magnesium, sodium, and potassium cations, carbonate, hydrogen carbonate, and chloride. The Saudi coastal waters of the Red Sea and the Arabian Gulf are characterized by significantly higher salinities and TDS concentrations than in other open seas. Normal sea-salinity is ≈35‰ and normal TDS concentrations are 25,000–35000 mg/l [15, 16]. Average TDS values recorded during the study were stable at ≈ 45,000 mg/l. Both salinity and TDS showed minimum variation between seasons because the temperature is conducive to evaporation in all seasons and precipitation is scarce. TDS fall within values typical of the Gulf coastal waters [12, 17].

High salinity and TDS are typical of Gulf water because of the restricted flow in the Straits of Hormuz, coriolis force and the high evaporation rates. These relatively high salinity and TDS values are significant in desalination economics because high levels of energy are required to extract product water using the desalination membranes. Energy recovery devices are usually installed in SWRO plants to reduce energy consumption, and this should also be the case for the proposed SWRO plant at this location.

### 3.7 Total hardness

Hardness is one of the key parameters affecting water recovery in both thermal and RO desalination. The total hardness was monitored, and total hardness values fell within the normal range of the Arabian Gulf with a range of 6648–8648 mg/l as CaCO_3_with averages of 7761, 8033, 8124, and 8172 mg/l as CaCO_3_ for summer, autumn, winter, and spring, respectively with no statistically significant differences between the seasons (Table 3). Seawater typically has a total hardness of ≈6630 mg/l as CaCO_3_ [18]. Elevated hardness in Gulf water requires the use of antiscalants to control scale formation on SWRO membranes.

### 3.8 Total organic carbon (TOC)

In general, there are no significant differences in TOC values between the seasons. Average TOC values were 2.37, 2.40, 2.30, and 2.10 mg/l for summer, autumn, winter, and spring, respectively (Table 3).

In addition to its inorganic major, minor and trace constituents, seawater contains both particulate and dissolved organic matter [19]. The total organic carbon (TOC) in water is composed of a variety of organic compounds in various oxidation states [5]. Some of these compounds can be oxidized further and used by various marine organisms. Others are refractive and cannot be used. TOC is a measure of all these carbon fractions in water and it is also a measure of the organic load in water rather than a nutrient index. From a summary of values in the literature, Williams [20] concludes that in shallow water (<100 m) dissolved organic carbon values range from 0.6 to 2.0 mg/l. The values reported in this study were close to the global range.

TOC values do not reflect the presence of oil as supported by concentrations of the trace metals vanadium and nickel (see section on Ions and tracemetals below). TOC values also do not reflect the presence of other pollutants like sewage (see section on Bacteriological analysis below).

### 3.9 Major ions and trace metals

The concentration of major ions is presented in Table 4 with reference concentrations reported for normal seawater [21, 22, 23, 24]. Their concentration in the sea is normally very stable. In the Gulf, concentrations are slightly higher than those concentration found in “normal” seas. This is because of the higher ionic concentration of Arabian Gulf water. The concentration of phosphate is similar to that reported in Gulf coastal water at Jubail [2], and it is lower than the values reported for the Al-Kkafji Joint Operation Concession area [1]. Since phosphorous is a major factor limiting phytoplankton growth, the present phosphorous concentration should not support massive algal blooming.

Trace metal concentrations (Table 5) fell within the ranges reported in the Arabian Gulf and in “normal” seas [21, 23, 24, 25, 26, 27, 28, 29]. Therefore, these concentrations do not indicate any type of pollution, particularly oil pollution. Of particular interest are nickel and vanadium, which are commonly associated with oil spills. For example, the Australian and New Zealand Environment and Conservation Council (ANZECC) set a trigger value for nickel and vanadium at 70.0 and 100.0 μ/l, respectively. The extremely low values reported here give rise to no concern. Boron concentration in the Gulf is slightly higher than that in seawater (Table 5). SWRO membranes reject 51–70% of boron, the reminder appearing in product water [26]. Assuming that 50% of boron content is rejected by SWRO membranes, product water would contain 2.3–2.6 mg/l boron (50% of concentrations reported in Table 5). The WHO sets the guideline value for boron in drinking water at 2.4 mg/l [30]. Given that SWRO membranes reject over 50% of boron, the present boron concentration in Khafji does not call for any special kind of treatment.

### 3.10 Chlorophyll-a

Chlorophyll is a reliable indicator of the productivity status of an aquatic environment. The synthesis of organic compounds from the inorganic constituents of seawater, known as ‘primary production’ is performed by chlorophyll-containing plants in the sea through the process of photosynthesis. The monthly level of Chlorophyll *a* production varied from 0.5 to 4 μg/l. The highest Chlorophyll *a* values occurred in fall and winter, and the lowest in summer. The mast important group of phytoplankton is diatoms, which are more reproductive in lower temperatures. Chlorophyll values were within the normal range for Gulf coastal waters [2, 27].

### 3.11 Dissolved oxygen and biochemical oxygen demand (BOD_5_)

Dissolved oxygen values were close to saturation levels (5.5–7.0 mg/l), indicting a well-mixed water column. Dissolved oxygen in the intake bay of the SWCC Jubail desalination and power plants (the bay is protected from powerful waves and wind action) ranged from 4.6 – 5.1 mg/l [2]. It was mentioned above the agitation of the water column results in a higher TSS load. Dissolved oxygen is near to saturation levels in surface water throughout most of the Gulf, ranging from 4.8 to 6.5 mg/l [12].

BOD values ranged from 0.2 to 1.9 mg/l with no significant seasonal differences. These values are low, indicating a lack of nutrient enrichment from external/pollution sources. The Presidency of Meteorology and Environmental Protection (PMEP) draft guidelines for BOD in marine water discharges is 15 mg/l water. The present value falls well below this limit.

### 3.12 Bacteriological analysis

The 0-h count ranged from n × 10^2^ to n × 10^3^ CFU/ml and the 48-h count averaged n × 10^5^ CFU/ml, where n is a single digit number, usually <5. The 48-h generation time ranged from 4.2 – 6.0 h. There were no coliform or fecal coliform bacteria detected at any sampling date.

Initial bacterial densities (0-h counts), which are in the order of n × 10^2^ to n × 10^3^, were lower than those densities in clean, pristine Gulf water. For example the densities for source water at the Jubail Desalination and Power Plants, south of the present site, were in the order of n × 10^4^ CFU/ml [5]. However, the 48-h counts were similar to those in Jubail coastal waters at n × 10^5^ CFU/ml. Bacterial growth rates at the site exceed those in Jubail. This is reflected in the 48-h doubling time (4.2–6.0 h) which is faster than that in Al-Jubail (12.5–20.0 h). This could be attributed to the lower starting counts at 0-h, or to the presence of more inorganic nutrients (NH_3_, NO_3_, and PO_4_) in Al-Khafji than in Al-Jubail water [2].

This does not mean that the accelerated bacterial growth rate at Al-Khafji would lead to more membrane fouling in the proposed SWRO plant. This is because membrane biofilm formation is not a product of mere bacterial cells, but rather a product of extracellular products secreted by bacteria and algae on the membrane or in source water. For example, hardly any bacterial isolates were implicated in biofilm formation on membranes at the SWCC Al-Jubail SWRO plant [31]. The formation of biofilm also needs the attachment surface to be conditioned by organic molecules prior to bacterial settlement. If the concentration of total organic carbon (TOC) is indicative of these conditioning molecules, then the concentration profile of the TOC will be comparable to that in pristine Gulf coastal waters and not indicative of eutrophication/pollution. However, the picture is different with chlorination. Chlorine could solubilize some refractive organic matter and thus provide conditioning surface for biofilm formation on membranes (see below).

### 3.13 Chlorination disinfection

Chlorination caused significant increases in SDI and dissolved carbohydrates and proteins concentrations (Table 6). Higher SDI probably resulted from breakdown by chlorine of larger particles and consequent plugging of the SDI filter paper pores (filter pore size was 0.45 μm). The increase in dissolved carbohydrates and proteins resulted from breakdown of some organic matter by chlorine. Soluble organic molecules could escape filtration and condition membranes surface for subsequent attachment of biofilm layers. Apparently, chlorination also stimulates aggradation of solitary bacteria into large masses that more readily attach to the membranes surface. With the exception of total suspended solids, chemical and physicochemical parameters measured in this study are typical of values reported for the Arabian Gulf waters and for sweaters in general.

Chlorination coupled with a high variability TSS load may result in filtrations problems and episodes of high SDI at the SWRO plant. The plant should be judicious and thoughtful about the application of chlorine. With sound pretreatment, the plant may need to use chlorine only to protect the intake structures from marine shell fouling. The mode of chlorination could be shock dozing, intermittent or pulse chlorination. Specifically, the plant is advised to employ an intermittent mode of chlorination to protect the intake structures from marine shell fouling. However, the plant is under commissioning now and is operating with continuous chlorine dosing at the intake pit. Chlorine is removed completely and is not allowed to reach the membranes at any time.

### 3.14 Current status of the plant

The plant adopted and tested a pretreatment method essentially same as suggested in the preceding discussion. As advised, the plant located its intake at deeper water 2,100 m off shore at a depth of approximately 10 m. The intake pit constitutes a fiberglass tower that allows water to flow under gravity from 2 m above the bottom through a 1.3 m-diameter pipe. The water seeps through the intake pipe to a receiving concrete tank on shore measuring 24 m × 10 m × 9 m. At the entrance to the tank there is a travelling screen to bar large debris access to the tank and is followed by a rotating drum screen of 2 mm-mesh size to remove smaller particles. The tank could be viewed as a sedimentation pond for removing suspended particles. Sedimentation could be aided by addition of a coagulant. The water from the tank is pumped to a dual media filter (DMF) followed by straining self-cleaning filter of 100 μm pore size, and then to an ultrafiltration unit. The straining filter acts as a micron cartridge filter, routinely employed in SWRO plants, to block large particles separating from the DMF access to the ultrafiltration unit. Chlorine is added continuously in the intake pit and residual chlorine is removed completely after the ultrafiltration unit and does not reach the membranes at all.

The plant design production capacity is 70,000 m^3^/day but the operation capacity is around 60,000 m^3^/d. The pretreatment system produces water of acceptable quality with SDI values of ≤1–2.5 after the ultrafiltration unit. However, SDI after the DMF ranges from 3.8 – 4.0. The plant uses spiral wound membranes with 15-min SDI limit of 3.5. In general, the SDI limit of seawater reverse osmosis membranes is met with a DMF. Because SDI of DMF filtrate is relatively high, the design of the DMF needs to be checked for appropriateness of media depth and grain size. Otherwise, the plant may face filtration problems and higher SDI values after ultrafiltration. It was stated in the discussion of turbidity and total suspended solids (Section 3.2) that variable suspended solid loads may create filtration problems for the plant. If the DMF produces water of acceptable SDI levels, the plant could have a provision to by-pass one of the filter units and operate on either the DMF or ultrafiltration. This would conserve pretreated water that is used in backwashing filters and reduce operation cost.

After 8-month operation, there in no increase in transmembrane pressure or deterioration in product quantity or quality. However, the plant is working at approximately 85% of its design capacity.

## 4 Conclusions

1. The Gulf is characterized by high salinity, high water temperatures that are conducive to biological growth, frequent encounters of natural phenomena (e.g. red tide and jellyfish swarms), shallow depth that makes water column easily disturbed, and extremely low water turnover. These characteristics pose distinct challenges to SWRO plants operation.
2. With the exception of total suspended solids, chemical and physicochemical parameters measured in this study are typical of values reported for the Arabian Gulf waters or for sweaters in general. High and variable TSS concentrations call for proper filtration regimen to avoid operational problems.
3. Most of beach lands are owned privately or publicly and SWRO plant owners do not have the luxury of choosing the most desired location for a SWRO plant. The owners must use land available to them. Nevertheless, with the proper pretreatment of intake water SWRO plants could successfully site and operated at sites of compromised water quality.

## Declarations

### Author contribution statement

Mohamed Saeed: Conceived and designed the experiments; Performed the experiments; Analyzed and interpreted the data; Contributed reagents, materials, analysis tools or data; Wrote the paper.

Mohamed Al-Nomazi, Ahmed Alamudi: Conceived and designed the experiments; Performed the experiments; Contributed reagents, materials, analysis tools or data.

### Funding statement

The authors received no funding from an external source.

### Competing interest statement

The authors declare no conflict of interest.

### Additional information

No additional information is available for this paper.
